# In-Source Decay and Pseudo-MS^3^ of Peptide and Protein Ions Using Liquid AP-MALDI

**DOI:** 10.1007/s13361-016-1511-0

**Published:** 2016-10-17

**Authors:** Rima Ait-Belkacem, Marialaura Dilillo, Davide Pellegrini, Avinash Yadav, Erik L. de Graaf, Liam A. McDonnell

**Affiliations:** 1 Fondazione Pisana per la Scienza ONLUS, Pisa, Italy; 2 Dipartimento di Chimica e Chimica Industriale, University of Pisa, Pisa, Italy; 3 Scuola Normale Superiore di Pisa, Pisa, Italy; 4 Center for Proteomics and Metabolomics, Leiden University Medical Center, Leiden, The Netherlands; 5 Department of Pathology, Leiden University Medical Center, Leiden, The Netherlands

**Keywords:** MALDI, In-source decay, MS/MS, Pseudo MS3, T3 sequencing

## Abstract

Atmospheric pressure MALDI on a Q-Exactive instrument was optimized for in-source decay and pseudo-MS^3^. The dependence of AP-MALDI ISD on the MALDI liquid matrix was investigated for peptides and proteins. The liquid matrices enabled long-life ISD signal, and exhibited high fragment ion yield and signal stability. Extensive a-, b-, c-, y-, and z-type fragment series were observed depending on the matrix used but were most extensive with 2,5-DHB. Complete sequence coverage of small peptide and intact protein-terminus sequence tags were obtained and confirmed using HCD as a pseudo-MS^3^ method.

**Electronic supplementary material:**

The online version of this article (doi:10.1007/s13361-016-1511-0) contains supplementary material, which is available to authorized users.

**Graphical Abstract:**
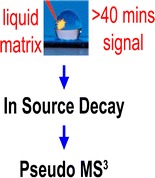
ᅟ

## Introduction

In-source decay (ISD) is the principal MS/MS method available to matrix-assisted laser desorption/ionization (MALDI) that can also be applied to intact proteins. In ISD high laser fluence is used to initiate a fragmentation process in the hot MALDI plume during analyte desorption/ionization. ISD fragment ions correspond mainly to *c-* and *z-*ions [1, 2], but other fragment ions such as *a*-, *b-*, and *y-*ions have also been reported [3–7]. The obtained fragments were strongly dependent on the matrix used, as well as the acidic and basic characters of the constituent amino acid residues and the near N- and C-termini [6]. The major drawbacks of ISD are the lack of precursor ion selection and the matrix cluster background that can obscure fragment ions in the lower mass range. The ultra-high mass resolution and high mass accuracy of Fourier transform mass spectrometry have been exploited to resolve more of the fragments [8]. Alternatively, a pseudo-MS^3^ method incorporating ISD-fragment-isolation and post-source decay (PSD) has been used to acquire sequence tags from specific ISD fragments, and therefore unencumbered by the matrix background.

The advantages of high mass resolution and high mass accuracy for protein identification are very well established, especially so for intact protein analysis. The Q-Exactive range of Orbitrap mass spectrometers (Thermo Scientific, Bremen, Germany) provide routine high mass resolution and high mass accuracy with higher energy collisional dissociation (HCD), but are only available with atmospheric pressure MALDI sources from third party vendors. AP-MALDI is generally considered to be a soft ionization technique with an upper mass limit of approximately 5 kDa. ISD is able to generate sequence information from proteins spanning a wide mass range, and preliminary data indicates this allows AP-MALDI to analyze intact proteins in spite of its limited mass range [9]. In addition to high mass resolution accurate mass ISD, HCD would also enable high mass resolution and accurate mass pseudo-MS^3^ sequencing.

MALDI sample preparations have a finite lifespan in that high intensity analyte ion signals are obtained only while sufficient matrix remains. The higher laser fluence used for ISD experiments exhaust typical MALDI matrices quickly. Longer life signals have been reported for MALDI matrix solutions incorporating nitrocellulose and liquid matrices [10–15]. In this study, we investigated the potential of high mass resolution, accurate mass ISD, and pseudo-MS^3^ using an AP-MALDI enabled Orbitrap and long-life MALDI matrices.

## Experimental

### Materials

Sinapinic acid (SA), α-cyano-4-hydroxycinnamic acid (α-CHCA), 2,5-dihydroxybenzoic acid (2,5-DHB), super-DHB, 1,5-diaminonaphthalene (1,5-DAN), bradykinin, thymosin β4 human recombinant, horse heart cytochrome *c*, ammonium sulfate, glycerol, methanol, and water LC-MS UltraChromasolv were purchased from Sigma-Aldrich Chemical Co. (St. Louis, MO, USA). SuperFROST microscope glass slides were from VWR International (Radnor, PA, USA).

### Sample Preparation

Bradykinin, thymosin β4, and cytochrome *c* were dissolved in Milli-Q (Watford, United Kingdom) water to a final concentration of 1 mg/mL, and then stored at – 20 °C until further use. For solid matrix preparations, α-CHCA and 2,5-DHB solutions were prepared at saturation in 70/30 (v/v) ACN/H_2_O 0.2% TFA. Liquid matrices were prepared by the addition of 100 mM ammonium sulfate/methanol (1:1; v/v) to UV-MALDI matrices (SA, α-CHCA, 2,5-DHB, super-DHB, and 1,5-DAN) in a ratio of 10:1; (v[μL]/w[mg]). Then, 20% glycerol was added and the mixture vortexed and then sonicated for 15 min [10]. One μL of analyte solution was mixed with 2 μL of matrix solution and then spotted on a superFROST microscope glass slide.

### AP-MALDI

All experiments were performed using a Q-Exactive Plus (Thermo Scientific, Bremen, Germany) equipped with a nitrogen laser based AP-SMALDI10 source (TransMIT GmbH, Giessen, Germany). ISD experiments were performed using elevated laser fluence, with on target laser energy of 3.5 to 10.5 μJ and laser spot size of 20 μm. The experiments were performed using the 2D Scan-Pixel Mode and each scan consisted of the accumulated ions from 30 laser pulses fired at 60 Hz. For maximum ion yield, the MALDI target was held at 4.0 to 4.3 kV, and the inlet temperature set to 250 °C. The Q-Exactive Plus was set for a maximum injection time of 500 ms, full scan mode was used, and each spectrum averaged 200 scans. Ions were detected in positive-ion mode, in the mass range 1000 to 5000 *m/z* and with a resolution of 140 k at *m/z* 200. For pseudo-MS^3^ analysis, ISD fragments were selected using an isolation window of 1.2 *m/z* then fragmented using HCD with a normalized collision energy of 35%.

### Peak Annotation

The ISD fragments correspond to N- or C-terminal fragments. Windows Prosight Light was first used to obtain an overview of the N- and C-terminal fragments (*b*, *c*, *y*, *z*). Fragment ion peaks were then assigned with ProteinProspector [16]. They included both internal and terminal types of fragmentation (*a*, *b*, *c*, *x*, *y*, *z*) including satellite sequences (side-chain loss generating *v*, *d*, *w* ions) as well as NH_3_ and H_2_O neutral losses. For pseudo-MS^3^ an ISD fragment was in-silico fragmented using ProteinProspector and MS^3^ fragment ions assigned using a 2 ppm mass tolerance.

## Results and Discussion

Here we investigated the analytical capability of AP-MALDI using long-life matrices to generate ISD and pseudo-MS^3^ fragments of peptides and proteins. The experiments were performed on a Q-Exactive Plus equipped with an AP-MALDI imaging source, which thus enabled accurate mass, high mass resolution characterization of the ISD, and pseudo-MS^3^ fragments. Thus, the method extends the high mass resolution, accurate mass ISD recently reported by Nicolardi et al. [8] to also include targeted ISD fragment sequencing and optimized liquid matrices.

The liquid matrix has already been investigated for diverse molecular classes with vacuum MALDI [11–15], but has not been thoroughly investigated for AP-MALDI, for which different matrix preparations have been found to be necessary [17, 18]. This is pertinent because the generation of ISD fragments and the sequence coverage obtained are highly influenced by the MALDI matrix and laser fluence [19–23]. Accordingly, different matrices, laser fluence, and analytes (peptide and proteins) were tested, including a variety of matrix additives to increase the durability of the MALDI sample, such as glycerol for liquid matrix [10, 24], and the sequestration of excess salts (ammonium sulfate [25]). By using a liquid matrix with added ammonium sulfate, our data showed a significant improvement compared with solid matrix, and the MS/MS spectra were more reproducible and with increased signal (Supplementary Figure 1).

**Figure 1:**
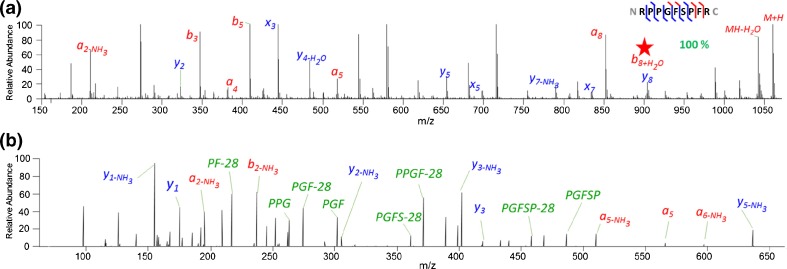
(**a**) AP-UV-MALDI ISD spectrum of bradykinin peptide using 2,5-dihydroxybenzoic acid (2,5-DHB) liquid matrix. (**b**) Pseudo-MS^3^ spectrum of *y*_*8*_ */b*_*8*_ + H_2_O fragment ion (indicated with a red star in the ISD spectrum). Annotated *a-*, *b-*, (red) and *x-*, *y-* (blue) ions with or without neutral losses and internal fragments (green) are shown, as well as the amino acid sequence highlighting the sequence coverage of 100%

The dependence of bradykinin peptide fragmentation to liquid matrix composition has been investigated. It was found that 2,5-DHB provided the greatest sequence information, which yielded the highest total ion count and complete sequence coverage among the (*a-*, *b-*, *y-*, and *x-*) fragment channels (Figure 1a). The other matrices, SA, 1,5-DAN, α-CHCA, and super-DHB generated less fragment ions and lower sequence coverage (50% with SA, 63% with 1,5-DAN, and 88% with α-CHCA) (Supplementary Figure 2).

The bradykinin ISD fragment ion detected at nominal mass *m/z* 904 can be one of two fragment ions of identical mass, a *y*_*8*_ fragment or a *b*_*8*_ + H_2_O fragment (*y*_*8*_, PPGFSPFR=C_44_H_62_N_11_O_10_: 904.4676 Da; *b*_*8*_+H_2_O, RPPGFSPF=C_44_H_62_N_11_O_10_: 904.4676 Da). To identify the fragment ion, it was isolated and fragmented with HCD. Figure 1b shows the pseudo-MS^3^ fragmentation that confirmed its identity as *b*_*8*_ + H_2_O with 75% sequence coverage.

**Figure 2:**
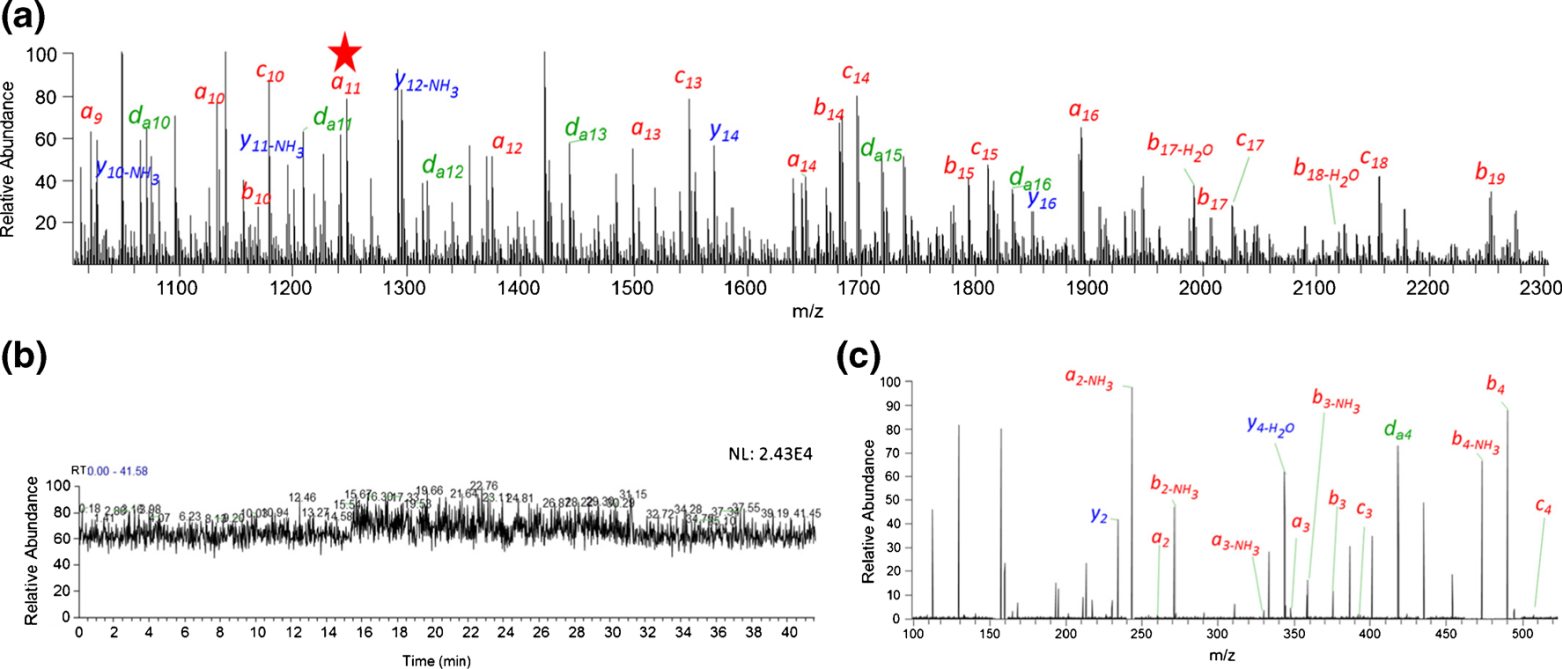
AP-UV-MALDI ISD spectrum (*m/z* 1000–2300) of thymosin β4 human recombinant protein using 2,5-dihydroxybenzoic acid (2,5-DHB) liquid matrix (**a**). *a-*, *b-*, *c-* (red), and *y-* (blue) with or without neutral losses, including side-chain loss generating *v-*, *d-*, *w-* ions (green) are labeled. (**b**) Total ion chromatogram (TIC) over 42 min data acquisition using 2,5-DHB liquid matrix mixed with 192 pmol thymosin β4. AP-UV-MALDI pseudo-MS^3^ spectra of *a*_*10*_ = 1113.49 thymosin β4 fragment ion (indicated with a red star in the ISD spectrum). Annotated *y-*ions (blue) and *a-*, *b-*ions (red) with or without neutral losses are indicated, including side-chain loss generated *d-*ions (green) (**c**)

AP-MALDI is generally used for the analysis of small molecules and peptides because of a drop-off in sensitivity at high mass. ISD can generate structurally informative fragments from larger proteins, and so allows the characterization also of peptides and proteins beyond its nominal mass range. Supplementary Figure 3 shows the dependence of AP-MALDI ISD of thymosin β4 on the liquid matrix formulation. Extensive sequence information was obtained with all liquid matrices; SA gave 62% sequence coverage, 1,5-DAN gave 52% sequence coverage, α-CHCA gave 64% sequence coverage, and 2,5-DHB 68% sequence coverage. All confirmed the amino acid sequence of this recombinant glycoprotein containing 45 amino acid residues (RMSDKPDMAEIEKFDKSKLKKTETQEKNPLPSKETIEQEKQAGES). Figure 2a shows that 2,5-DHB visually provided more fragments (*m/z* 1000–2300) and at higher average signal intensity, with extensive *a-* and *c-*fragment ion series and additional high-energy CID-like related satellite ions (*d-*ions), as has been previously reported for vacuum ISD [6]. The *a-*fragments are formed by the loss of CO- from *b* series fragments under the higher laser fluence needed for this matrix [26]. The 2,5-DHB liquid matrix provided a durable and stable ion yield and signal (Figure 2b), enabling many individual scans to be accumulated, important for automated pseudo-MS^3^ of larger proteins. Figure 2c shows the pseudo-MS^3^ spectrum of *a*_*10*_ ISD fragment, in which very high *S/N* of many fragments were obtained after summing 1900 microscans.

**Figure 3:**
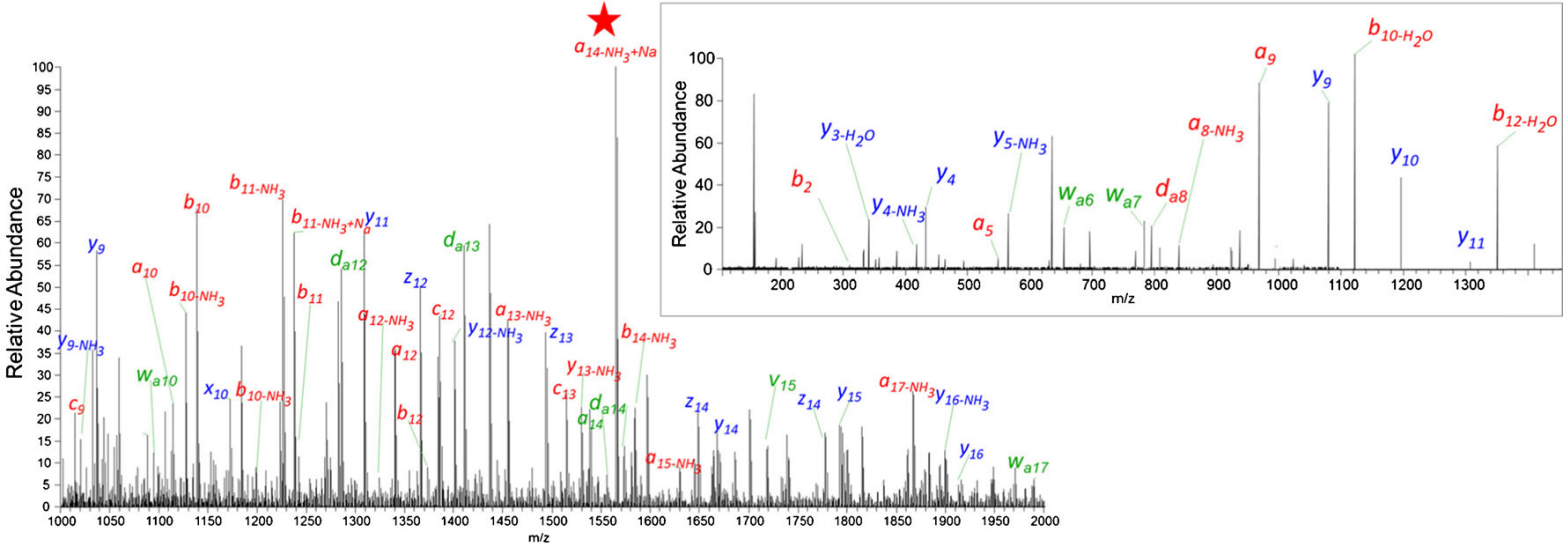
AP-UV-MALDI ISD spectra of cytochrome *c* protein using 2,5 DHB liquid matrix. The insert shows AP-UV-MALDI pseudo-MS^3^ spectrum of the *a*_*13*_ *-*NH_3_ + Na ISD fragment (indicated with a red star in the ISD spectrum) with different product ions; *a-*, *b-*, *c-* (red) and x-, *y-*, *z-* (blue) with or without neutral losses ion fragments, including side-chain loss generated *v-*, *d-*, *w-*ions (green) are labeled

AP-MALDI ISD was also used to characterize even larger proteins. Forty percent sequence coverage was obtained from cytochrome *c* (detected mass range *m/z* 1000–2000) using liquid 2,5-DHB matrix (Figure 3). The identified sequence showed a removed initiator methionine, *N*-acetylglycine at position 2 and *N*-acetyl-lysine at position 100. The *N*-acetylglycine at position 2 was also confirmed by pseudo-MS^3^ of an *a*_*14*_ ISD fragment ion (Figure 3 insert), and the resulting ions were predominantly *a-*, *b-*, *y-* with neutral losses and satellites *w-* and *d-*ions (shown in insert).

Two principal ISD fragmentation pathways have been described [22, 23], (1) a radical-induced pathway activated by matrices with high reductive ability such as 1,5-DAN, and which leads to *c-*, *z-*, *w-*, and *d-*fragment ions; and (2) a thermally activated pathway activated by matrices with high proton affinity such as 2,5-DHB, super-DHB, and α-CHCA, and which leads to *y-*, *b-*, and *a-*CID-like ions. Within this mechanistic description, the AP-MALDI ISD results using liquid matrices indicate that the matrices that gave the most extensive sequence information, 2,5-DHB and α-CHCA, followed the ergodic fragmentation pathway via thermal activation. The rapid collisional cooling afforded by the atmospheric conditions also leads to lower energy fragmentation pathways even for matrices that characteristically provide high energy CID fragments with vacuum MALDI (e.g., DAN matrix, Supplementary Figure 3).

## Conclusions

This study demonstrated the clear advantage of using liquid matrix sample preparations for AP-MALDI ISD and AP-MALDI pseudo-MS^3^ for the characterization of peptides and proteins. Enhanced ionization efficiency and signal durability were noted, enabling the pseudo-MS^3^ characterization of multiple ISD fragments from a single spot. The highest sequence coverage was obtained with 2,5-DHB liquid matrix, which provided up to 100% sequence coverage for peptides, 68% for the small protein thymosin β4, and 40% for cytochrome *c*—for the latter, the limited *m/z* range of the AP-MALDI source limits the observable ISD fragments to N- and C-termini. These results demonstrate ISD and pseudo-MS^3^ sequencing is a generic process that can be performed on different instruments regardless of source pressure. The lower mass range of the AP-MALDI source limits ISD and pseudo-MS^3^ to small proteins, but both analyses benefit from the accurate mass and high mass resolution of the Orbitrap mass analyzer. Conversely, the much wider mass range of a vacuum MALDI TOF instrument can be used to acquire ISD spectra from large proteins, including antibodies [27], and pseudo-MS^3^ spectra from smaller ISD fragments, albeit with significantly lower mass resolution and mass accuracy. It should be noted that the liquid matrix is less applicable to vacuum MALDI and intermediate pressure MALDI systems that require an extraction field in the MALDI source because the liquid matrix droplet distorts the electric field lines.

## Electronic supplementary material

Below is the link to the electronic supplementary material.

ESM 1 (DOCX 842 kb)
