# Supplementary material for: In-Source Decay and Pseudo-MS3 of Peptide and Protein Ions Using Liquid AP-MALDI
Source: J Am Soc Mass Spectrom. 2016 Oct 17;27(12):2075–9. doi: 10.1007/s13361-016-1511-0 (PMC5088222; doi:10.1007/s13361-016-1511-0)
Supplement: Supplementary file 1 — (DOCX 842 kb) [file 13361_2016_1511_MOESM1_ESM.docx]

**In-Source Decay and Pseudo-MS^3^ of Peptide and Protein Ions Using Liquid AP-MALDI**

Rima Ait-Belkacem,^a^ Marialaura Dilillo,^a^ Davide Pellegrini,^a^ Avinash Yadav,^a^ Erik L. de Graaf,^a^ Liam A. McDonnell^a,b^

a) Fondazione Pisana per la Scienza ONLUS, Pisa, Italy

b) Leiden University Medical Center, Leiden, The Netherlands

*liam@fondazionepisanascienza.org*

Corresponding author and reprint requests:

Dr. Liam A. McDonnell

Fondazione Pisana per la Scienza ONLUS

Via Panfilo Castaldi 2

56121 Ospedaletto

Pisa, Italy

E-mail: liam@fondazionepisascienza.org

Tel: +39 050 974 061

Fax +39 050 9656178


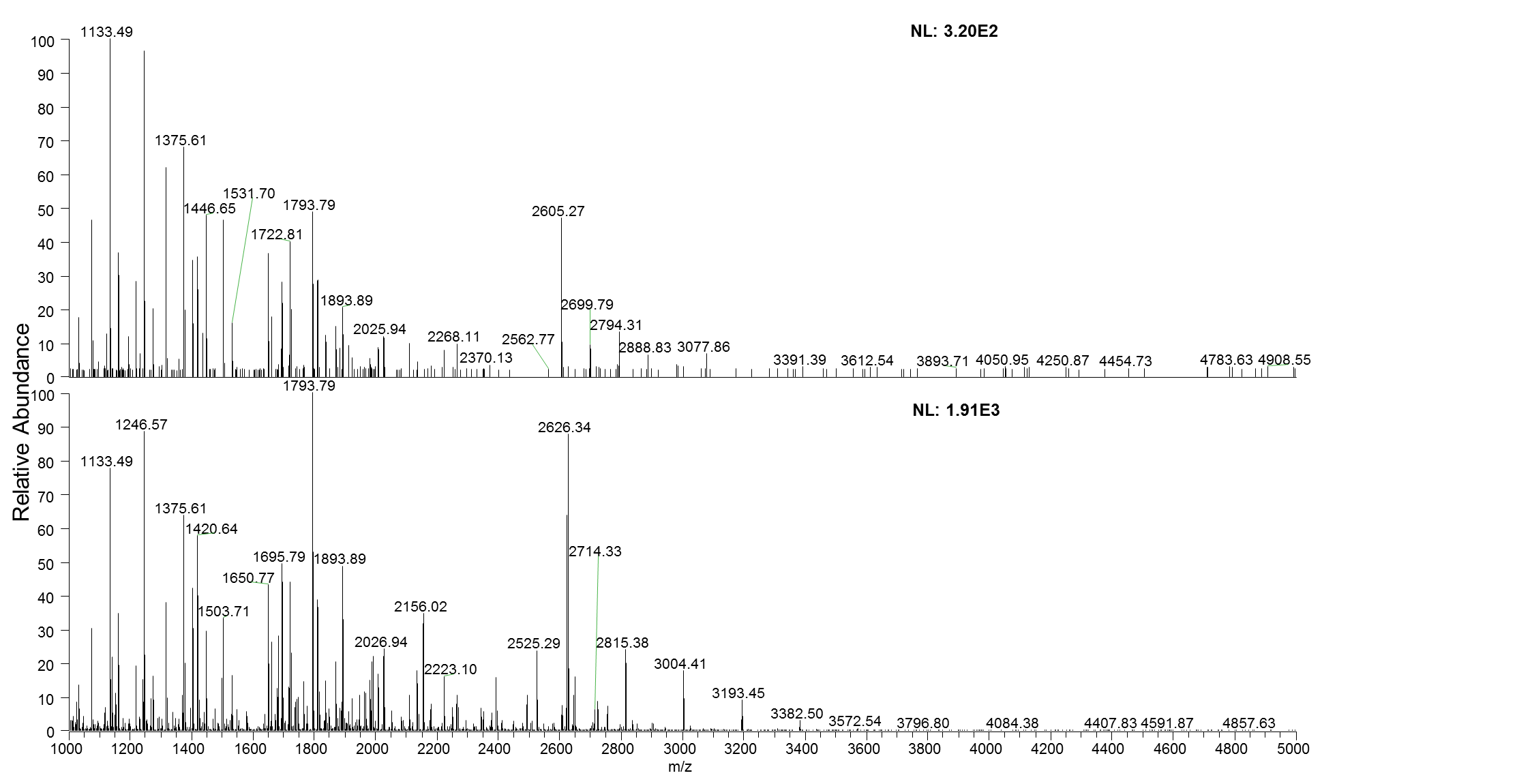

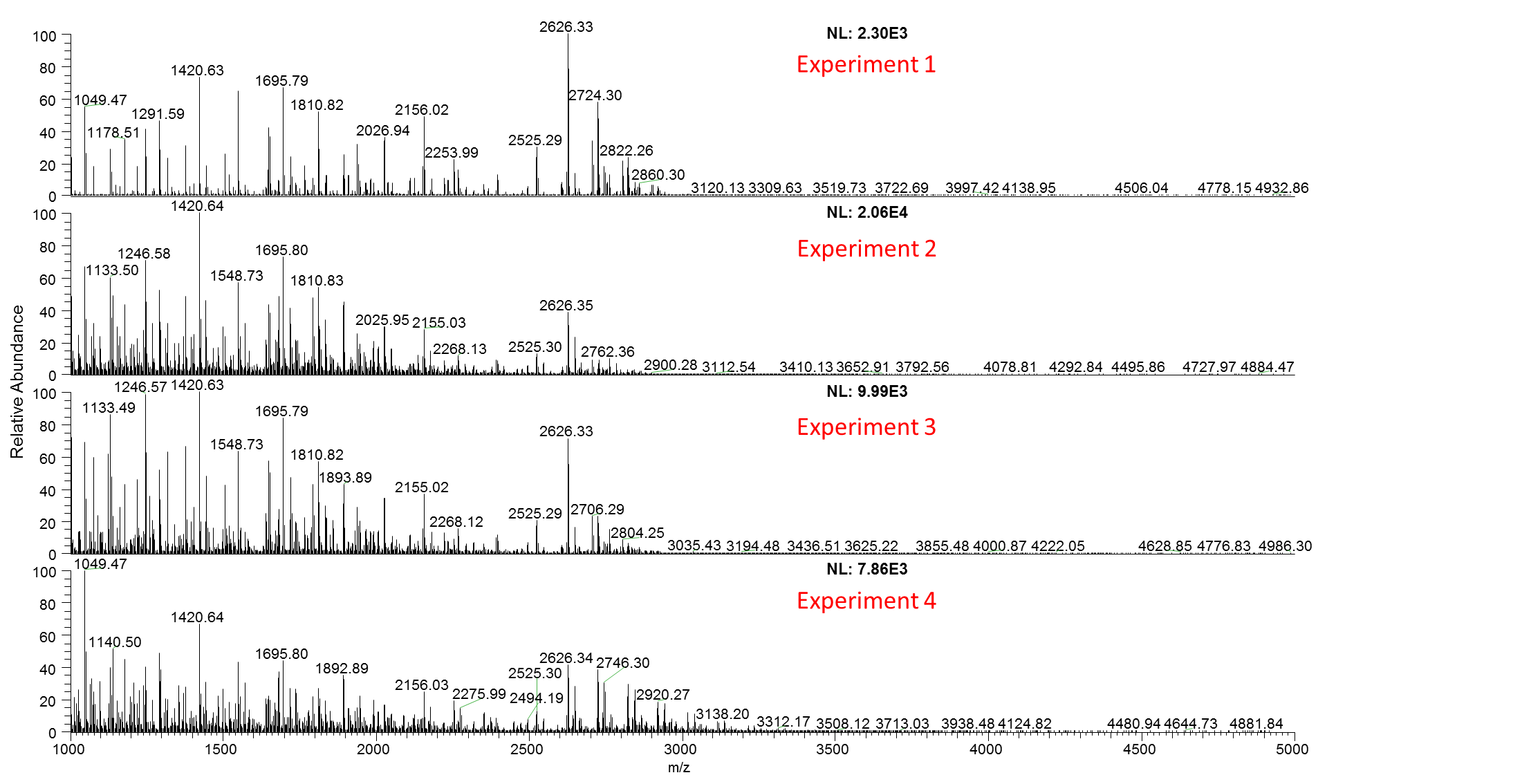


**Liquid matrix**

**Solid matrix**

**a)**

**b)**

**Supplementary figure 1.** Signal intensity increase of thymosin β4 human recombinant protein fragmentation efficiency and reproducibility with decrease of signal to noise level are noticed with all liquid matrices added ammonium sulfate. Solid and liquid preparations of 2,5-DHB matrix were performed and spectra are represented in (**a**). Different experiments of the same mixture (2,5-DHB liquid matrix/192 pmol of thymosin β4) were performed and reproducible spectra are presented in (**b**).


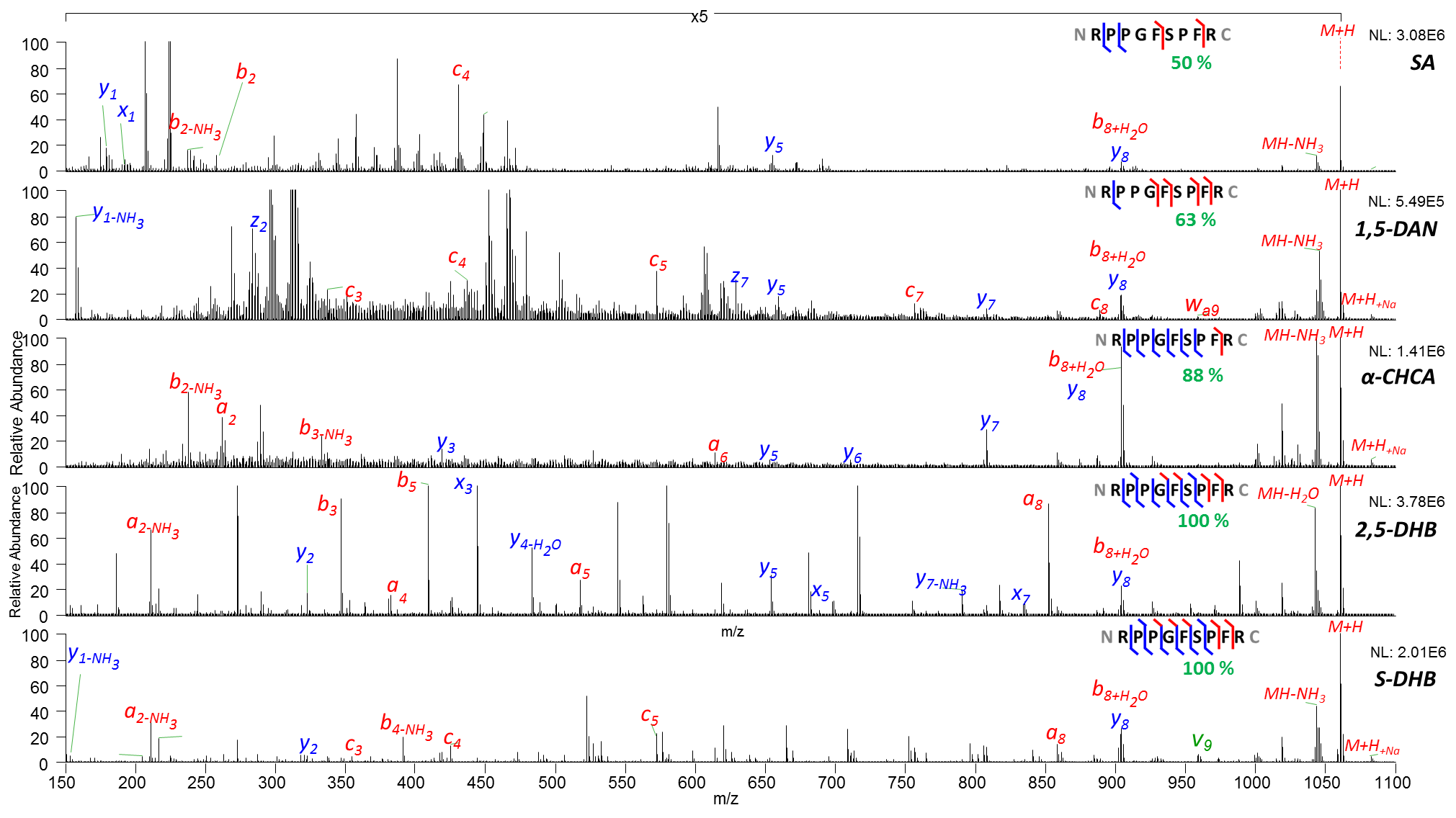


**Supplementary figure 2.** AP-UV-MALDI ISD spectra (at x5 zoom) of bradykinin peptide using sinapinic acid SA; 1,5-diaminonaphthalene (1,5-DAN); α[-cyano-4-hydroxycinnamic acid](http://www.sigmaaldrich.com/catalog/product/aldrich/476870?lang=en&region=US) (α-CHCA); [2,5-dihydroxybenzoic acid](http://www.sigmaaldrich.com/catalog/product/sigma/85707) (2,5-DHB) and super-DHB liquid matrices (from top to bottom) liquid matrices added 20% glycerol. Selected *a-, b-, c-* (red) and *y-* (blue) ion fragments are labeled clearly. The amino acid sequence highlighting the sequence coverage is also also provided


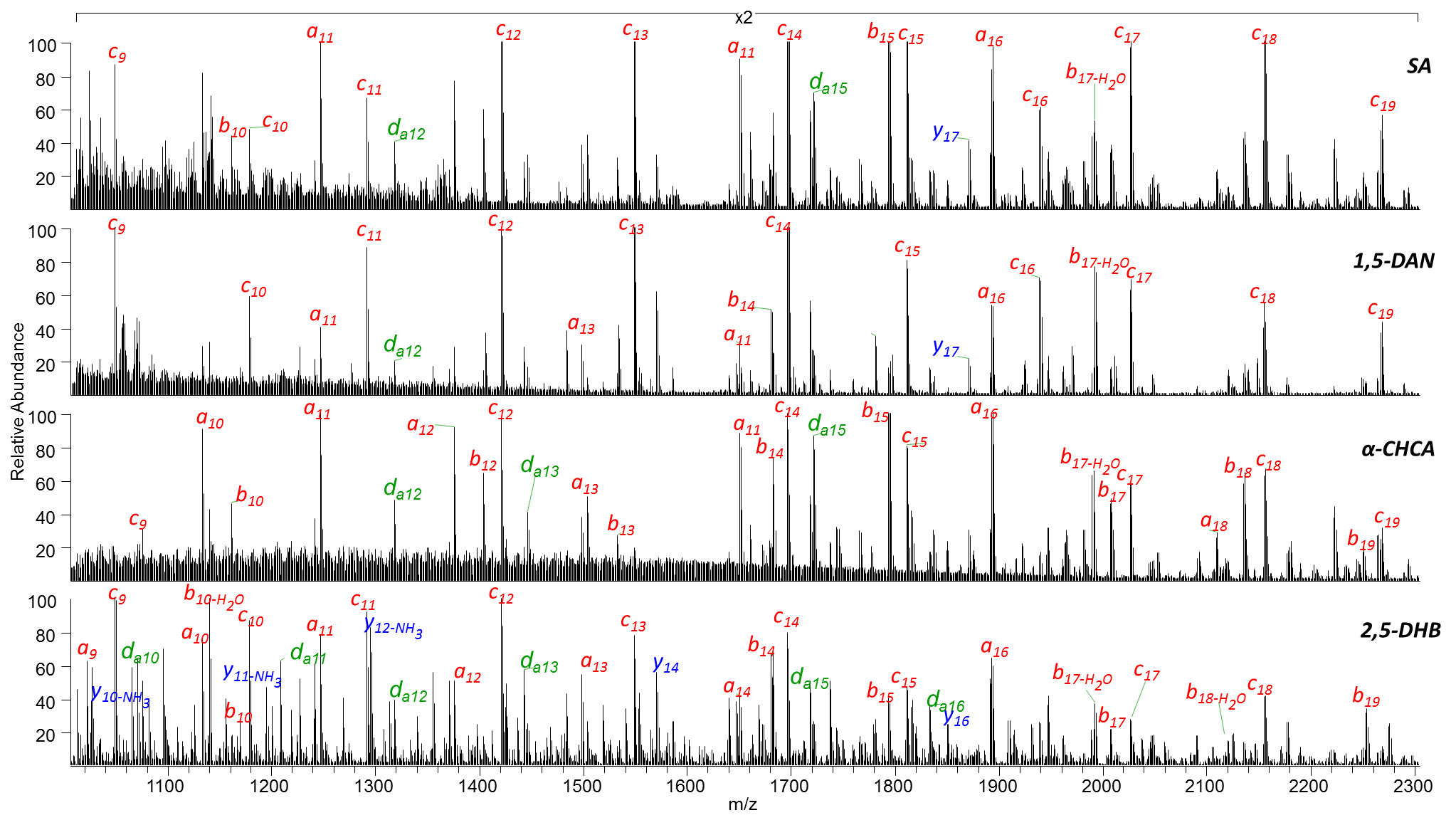

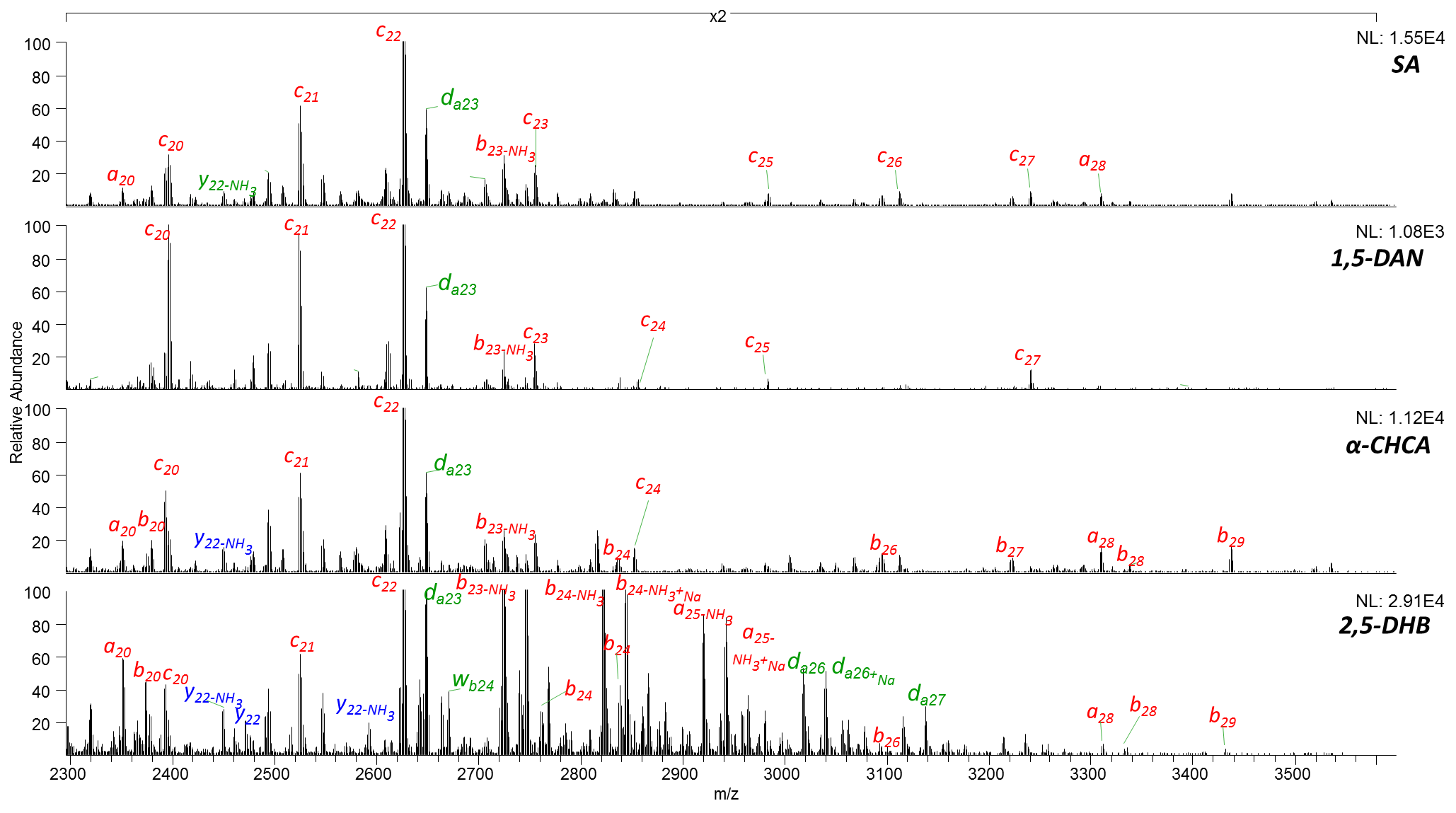


**Supplementary figure 3.** AP-UV-MALDI ISD spectra of thymosin β4 human recombinant protein using sinapinic acid (SA); 1,5-diaminonaphthalene (1,5DAN); α[-cyano-4-hydroxycinnamic acid](http://www.sigmaaldrich.com/catalog/product/aldrich/476870?lang=en&region=US) (α-CHCA) and [2,5-dihydroxybenzoic acid](http://www.sigmaaldrich.com/catalog/product/sigma/85707) (2,5-DHB) liquid matrices added 20% glycerol (from top to bottom). Selected *a-, b-, c-* (red) and *y-* with or without neutral losses (blue) ion fragments, including side-chain loss generating *v-, d-, w-* ions (green) are labeled
